# Acute Stanford Type A Aortic Dissection Mimicking Acalculous Cholecystitis: A Case Report

**DOI:** 10.7759/cureus.106022

**Published:** 2026-03-28

**Authors:** Mohammed Naji AlAbsi, Ganna A Ibrahim Sabri, Khaled F Alqudah, Mohammed Elkhazendar, Ahmad H Daraghmeh

**Affiliations:** 1 Department of Education, Sheikh Khalifa Medical City, Abu Dhabi, ARE; 2 Faculty of Medicine, The University of Jordan, Amman, JOR; 3 Department of Internal Medicine, Sheikh Khalifa Medical City, Abu Dhabi, ARE; 4 Department of Cardiology, Sheikh Khalifa Medical City, Abu Dhabi, ARE; 5 Department of Cardiology, SSM Health St. Clare Hospital, Fenton, USA

**Keywords:** acalculous gallbladder, atypical presentation of aortic dissection, cardiac tamponade, echocardiogram (echo), type a aortic dissection

## Abstract

Acute aortic dissection (AAD) is a time-critical cardiovascular emergency that classically presents with abrupt chest or back pain; however, atypical presentations may lead to misdiagnosis, delayed treatment, and worse outcomes. We report a case of AAD presenting with isolated abdominal pain and ultrasound findings mimicking acalculous cholecystitis. A 53-year-old male patient with poorly controlled hypertension and hypercholesterolemia presented with acute-onset epigastric and right upper quadrant pain associated with nausea. Lab evaluation showed direct bilirubin elevation and mild acute kidney injury. Abdominal ultrasound demonstrated gallbladder wall thickening and pericholecystic fluid without gallstones, raising a provisional concern for acalculous cholecystitis. ECG and initial troponin were unremarkable; however, serial troponin elevations prompted further assessment with transthoracic echocardiography (TTE), revealing an intimal flap in the ascending aorta with circumferential pericardial effusion. CT angiography confirmed Stanford type A dissection extending into the infrarenal abdominal aorta, associated with hemopericardium, inferior vena cava reflux, and preserved celiac perfusion. Emergent surgical repair demonstrated a contained rupture with sanguineous pericardial effusion consistent with low-grade tamponade. AAD may therefore present atypically with isolated abdominal symptoms driven by early tamponade physiology and venous congestion rather than visceral malperfusion. In patients with abrupt-onset pain and unexplained serial troponin elevations, timely consideration of dissection is paramount, even in the absence of chest pain.

## Introduction

Acute aortic dissection (AAD) is a life-threatening vascular emergency with an estimated annual incidence of 4.8 cases per 100,000 persons [1]. Stanford type A dissections involve the ascending aorta and account for approximately two-thirds of cases, while type B dissections, which spare it, comprise roughly one-third. Classical risk factors include hypertension, advanced age, connective tissue disorders, and bicuspid aortic valve [2,3]. AAD classically presents with abrupt, tearing chest or back pain; however, up to 10-15% of cases have painless or atypical presentations, including syncope, focal neurological deficits, limb weakness, and abdominal pain [4]. We report a case of AAD without chest pain mimicking acalculous cholecystitis.

## Case presentation

A 53-year-old male patient with poorly controlled hypertension and untreated hypercholesterolemia presented with a sudden onset of severe epigastric and right upper quadrant pain beginning shortly after a meal, associated with nausea. The pain was continuous, burning, and nonradiating. He denied fever, chest pain, back pain, vomiting, or dyspnea. He was physically active, a lifelong nonsmoker, and his only home medication was amlodipine.

Initial vital signs were significant for elevated blood pressure of 167/96 mmHg, heart rate of 63 beats per minute, and oxygen saturation of 100% on room air. Abdominal examination revealed epigastric and right upper quadrant tenderness with a negative Murphy’s sign. The remainder of the physical examination was unremarkable.

**Figure 1:**
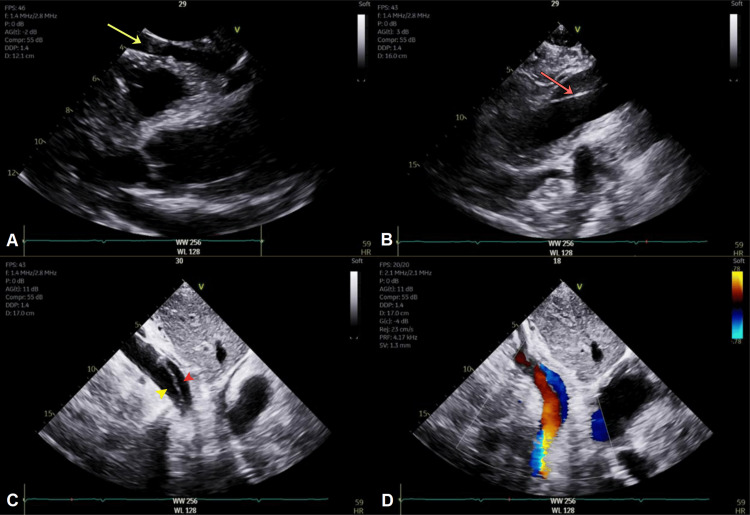
Transthoracic echocardiography demonstrating extensive Stanford type A aortic dissection (A) Zoomed-in parasternal long-axis view shows a normal-sized aortic root with moderate circumferential pericardial effusion (yellow arrow). (B) More superiorly, a mobile intimal flap (red arrow) is visualized within the dilated ascending aorta with a maximum diameter of 4.7 cm. (C) Abdominal aortic view demonstrates separation of the lumen into distinct true (yellow arrowhead) and false (red arrowhead) channels. (D) Color Doppler imaging reveals differential flow between the two lumens, further supporting the diagnosis. Left ventricular systolic function was preserved at 60-65%, with mild aortic regurgitation (not shown)

Initial differential diagnoses included hepatobiliary pathology and acute coronary syndrome. Laboratory evaluation (Table 1) revealed leukocytosis, mildly elevated aspartate aminotransferase (AST) (54 IU/L), direct bilirubin (8.6 micromol/L), and creatinine (115 micromol/L), with a normal initial troponin T (13.7 ng/L). Abdominal ultrasound demonstrated gallbladder wall thickening with pericholecystic fluid, without gallstones or biliary dilatation, findings initially supporting suspected acalculous cholecystitis. Serial troponins obtained because of persistent severe pain demonstrated a rise to 20.4 ng/L and 32.4 ng/L at two and eight hours, respectively, with unremarkable serial ECGs. This prompted further evaluation by echocardiography, revealing a type A aortic dissection extending into the descending thoracic aorta, associated with mild aortic regurgitation and moderate circumferential pericardial effusion, with otherwise preserved left ventricular function (Figure 1).

**Table 1:** Laboratory investigations upon admission and serial troponin-T levels CBC: complete blood count; RBC: red blood cell; MCV: mean cell volume; MCH: mean cell hemoglobin; MCHC: mean cell hemoglobin concentration; WBC: white blood cell; Na: sodium; K: potassium; Cl: chloride; CO_2_: Carbon dioxide (serum bicarbonate); ALP: alkaline phosphatase; AST: aspartate aminotransferase; ALT: alanine aminotransferase; PT: prothrombin time; PTT: partial thromboplastin time; INR: international normalized ratio;  pCO_2_: partial pressure of carbon dioxide; pO_^2^_: partial pressure of oxygen; HCO_3_: bicarbonate

Investigation	Value	Normal value
CBC		
RBC	5.13 x 10^6^/µL	4.20-5.60 x 10^6^/µL
Hemoglobin	16.1 g/dL	13.1-17.2 g/dL
Hematocrit	0.47 L/L	0.39-0.50 L/L
MCV	91.4 fL	80.0-100.0 fL
MCH	31.4 pg	27.00-34.00 pg
MCHC	34.30 g/dL	32.00-36.00 g/dL
Platelets	174 x 10^3^/µL	140-400 x 10^3^/µL
WBC	13.7 x 10^3^/µL (high)	4.5-11.0 x 10^3^/µL
Neutrophils	11.45 x 10^3^/µL (high)	1.80-7.70 x 10^3^/µL
Lymphocyte	1.08 x 10^3^/µL	1.50-4.00 x 10^3^/µL
Monocyte	1.11 x 10^3^/µL	0.20-0.95 x 10^3^/µL
Eosinophil	0.00 x 10^3^/µL	0.00-0.70 x 10^3^/µL
Basophil	0.03 x 10^3^/µL	0.00-0.15 x 10^3^/µL
Chemistry		
Na^+^	131 mmol/L (low)	136-145 mmol/L
K^+^	4.50 mmol/L	3.20-5.50 mmol/L
Cl^-^	98 mmol/L	98-107 mmol/L
CO_2_	19 mmol/L (low)	22-29 mmol/L
Anion gap	14 mEq/L (high)	8-16 mEq/L
Urea level	6.43 mmol/L	2.80-8.10 mmol/L
Creatinine	115 µmol/L (high)	62-106 µmol/L
Glucose	11.3 mmol/L (high)	3.9-6.0 mmol/L
Albumin	36 g/L	35-52 g/L
Lipase	21 IU/L	13-60 IU/L
ALP	60 IU/L	40-129 IU/L
AST	54 IU/L	0-40 IU/L
ALT	32 IU/L	0-40 IU/L
Total bilirubin	22.9 µmol/L (high)	0.0-21.0 µmol/L
Direct bilirubin	8.6 µmol/L (high)	0.0-5.0 µmol/L
PT/PTT/INR		
PT	18.8 sec (high)	11.70-15.30 sec
PTT	34.40 sec	26.70-43.90 sec
INR	1.5 (high)	0.8-1.2
Arterial blood gases (ABG)		
pH	7.37	7.35-7.45
pCO_2_	34.7 mmHg (low)	35.0-45.00 mmHg
pO_2_	169 mmHg (high)	80-100 mmHg
HCO_3_	20 mEq/L (low)	22-26 mEq/L
Lactic acid	2.3 mmol/L (high)	0.5-2.2 mmol/L
Cardiac biomarkers		
Troponin T (0 hours)	13.700 ng/L	0.000-14.000 ng/L
Troponin T (2 hours)	20.400 ng/L (high)	0.000-14.000 ng/L
Troponin T (8 hours)	32.400 ng/L (high)	0.000-14.000 ng/L

**Figure 2:**
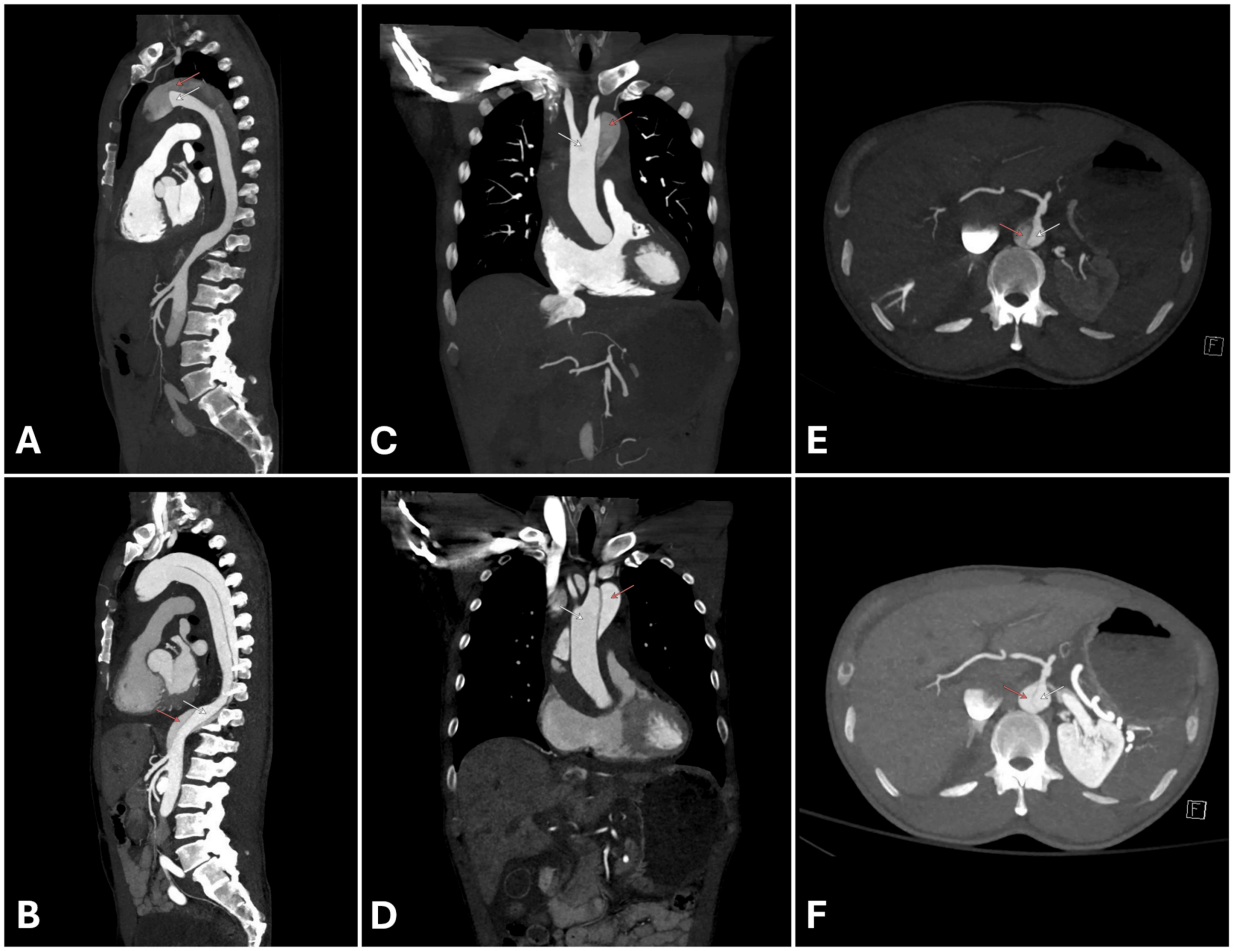
Contrast-enhanced CT angiography demonstrating Stanford type A aortic dissection Early (A) and late (B) arterial-phase sagittal multiplanar reconstructions demonstrate an intimal flap originating in the ascending aorta and extending through the aortic arch and descending thoracic aorta into the infrarenal abdominal segment. Late-phase imaging further delineates the separation of the true (white arrow) and false (red arrow) lumens. Early (C) and late (D) arterial coronal reconstructions show dissection of the ascending aorta and arch with differential luminal opacification. Reflux of contrast into the inferior vena cava is present, consistent with elevated right-sided pressures. (E) Axial imaging at the level of the celiac axis demonstrates the dissection flap adjacent to the celiac origin, with preserved opacification of the vessel. (F) Corresponding late arterial axial imaging further highlights the distinction between the true (white arrow) and false (red arrow) lumens

Subsequent CT angiography (Figure 2) confirmed Stanford type A dissection extending from the ascending aorta to the infrarenal abdominal aorta, with partial thrombosis of the proximal false lumen. The dissection flap abutted the celiac origin; however, the celiac trunk remained patent with preserved opacification. The right renal artery arose from the false lumen and the left from the true lumen, with preserved bilateral renal enhancement. A 1.3 cm high-attenuation pericardial effusion consistent with hemopericardium was present, along with pericholecystic free fluid. Reflux of contrast into the inferior vena cava and prominent hepatic veins were also noted, suggestive of elevated right-sided pressures.

Upon diagnosis, immediate blood pressure-lowering measures were necessary, along with transfer to a tertiary care center for emergent surgical repair. Following administration of a low dose of intravenous labetalol, the patient developed transient hypotension necessitating discontinuation of the medication. The patient underwent emergent ascending aortic and hemiarch replacement under cardiopulmonary bypass with deep hypothermic circulatory arrest. Intraoperative findings revealed a contained rupture with sanguineous pericardial effusion consistent with low-grade cardiac tamponade and a large intramural hematoma with a retroaortic leak. The aortic root and valve were preserved because aortic root dimensions and valve competence were normal, and no root tear was identified intraoperatively.

Following an uneventful intraoperative and postoperative course, the patient was weaned from bypass without mechanical circulatory support and extubated early postoperatively. A postoperative echocardiogram demonstrated mildly reduced left ventricular systolic function (ejection fraction 47 ± 5%), moderate right ventricular dysfunction, and moderate functional tricuspid regurgitation, without peri-graft leak or pericardial effusion. The patient underwent advanced physiotherapy and was discharged on day five. At subsequent outpatient follow-up visits, the patient remained asymptomatic with no angina, dyspnea, or peripheral edema.

## Discussion

AAD is a high-mortality cardiovascular emergency in which timely recognition is critical. Diagnostic delay, often driven by heterogeneous or atypical presentations, remains a major contributor to adverse outcomes. Untreated Stanford type A dissections carry a mortality of 1-2% per hour, and in-hospital mortality has improved in recent years; however, it remains substantial at approximately 22% despite advances in surgical therapy [2,3,5]. This case illustrates an atypical presentation of AAD with isolated abdominal pain and hepatobiliary imaging findings that obscured the diagnosis of AAD. Although abdominal pain may occur in dissection due to mesenteric ischemia or renal malperfusion, presentation as isolated gallbladder wall edema and right upper quadrant pain appears to be rare. A retrospective analysis of AADs identified an initial misdiagnosis in 51 of 361 patients; notably, only three patients were initially admitted with a diagnosis of acute cholecystitis [6].

Serial troponin elevations serve as a key diagnostic pivot, prompting transthoracic echocardiography (TTE) that identified the dissection. Troponin elevation in AAD represents a recognized diagnostic pitfall and may reflect coronary ostial involvement, myocardial ischemia, increased wall stress, or secondary myocardial injury. Importantly, misinterpretation as ACS may result in inappropriate antiplatelet or anticoagulant therapy with catastrophic consequences. In a prior cohort, 39.5% of patients with type A dissection had elevated troponin I, and these patients exhibited a higher 30-day mortality compared with their troponin-negative counterparts [7].

While TTE is a valuable early bedside screening tool, its pooled sensitivity for direct visualization of an intimal flap in type A dissection is approximately 89% (95% CI: 82-94%), emphasizing the need for CT angiography, which remains a definitive diagnostic modality which reduces diagnostic delay and delineates the extent of dissection [3,8]. Surgical repair remains the cornerstone of management. Prompt recognition and transfer in this case were critical to achieving a favorable outcome [3].

Contrast imaging in this case demonstrated hemopericardium with contrast reflux into the inferior vena cava and prominent hepatic veins, suggesting elevated right-sided pressures and evolving tamponade physiology. The transient hypotension following minimal beta-blockade was consistent with limited compensatory reserve and preload dependence in the setting of hemopericardium. The gallbladder wall edema and right upper quadrant free fluid were likely secondary to hepatic and splanchnic venous congestion rather than primary inflammatory disease, supported by inferior vena cava reflux, prominent hepatic veins, negative Murphy's sign, absence of fever, and rapid postoperative resolution. Furthermore, the preserved opacification of the celiac artery argues against primary arterial malperfusion, while propagation of the dissection to the renal arteries, combined with hemodynamic compromise, likely contributed to renal hypoperfusion and acute kidney injury [9].

Despite the abrupt pain, this patient would have been classified as low risk by the Aortic Dissection Detection Risk Score (ADD-RS) endorsed by the 2022 American College of Cardiology/American Heart Association (ACC/AHA) guideline for the diagnosis and management of aortic disease [4]. None of the high-risk conditions or physical exam findings were present, and his pain was not classical for dissection. Hence, this case highlights the limitations of ADD-RS in atypical presentations, where reliance on structured tools alone may delay diagnosis. Clinical judgment remains essential, particularly in patients with unexplained persistent pain or biomarker elevations. A similar framework is endorsed by the European Society of Cardiology (ESC) guidelines, which employs a similar risk stratification system. The ESC guidelines do, however, suggest that point-of-care ultrasound may be reasonably considered for selected low-risk patients [10]. D-dimer testing, which has high negative predictive value in low-risk patients, was not obtained because dissection was not initially suspected [4,10]. Whether D-dimer testing would have expedited diagnosis remains speculative.

## Conclusions

AAD may present with isolated abdominal pain and nonspecific hepatobiliary findings, including gallbladder edema related to early tamponade physiology and venous congestion, creating substantial diagnostic uncertainty. This case sheds light on the heterogeneous clinical presentation of AAD and underscores the limitations of structured risk stratification tools in atypical presentations. Clinicians should maintain a high index of suspicion in hypertensive patients with abrupt-onset pain and unexplained troponin elevations, even in the absence of classical chest or back pain. Despite a low ADD-RS risk classification, serial troponin assessment may alter diagnostic direction and expedite definitive imaging. Therefore, such tools should complement, rather than replace, clinical judgment. Early multimodality imaging remains essential to prevent catastrophic outcomes.
